# Central gland detachment following prostate artery embolization: incidence, risk factors and clinical outcomes

**DOI:** 10.1186/s42155-026-00774-9

**Published:** 2026-09-19

**Authors:** Fergus O’Herlihy, Sean Collins, Matthew Laffey, Kiaran O’Malley, Richard E. Power, Cormac Farrelly

**Affiliations:** 1 Department of Radiology, Mater Misericordiae University Hospital, Dublin, Ireland; 2 Department of Urology, Mater Misericordiae University Hospital, Dublin, Ireland; 3 Department of Urology, Beaumont Hospital, Dublin, Ireland; 4 Royal College of Surgeons Ireland (RCSI), Dublin, Ireland

**Keywords:** Prostate artery embolisation, Benign prostatic hyperplasia, Central gland detachment, Incidence, Risk factors

## Abstract

**Background:**

Central gland detachment (CGD) is a potential complication following prostate artery embolisation (PAE). There is limited information regarding its incidence, associated risk factors, and voiding and ejaculatory function after CGD.

**Methods:**

Sixty-five consecutive patients undergoing PAE in a single institution were included in this retrospective cohort study. Baseline demographics, radiologic, procedural and clinical outcome data were gathered for all patients. CGD was diagnosed following clinical review and confirmed via cystoscopy or MRI as required.

**Results:**

Six out of 65 (9.2%) patients undergoing PAE developed CGD. Mean baseline prostate volume was 150 mL. CGD occurred more frequently among patients with prostate volume > 150 mL than among those with volume ≤ 150 mL (19.2% vs. 2.6%, *p* = 0.03). Embolisation was performed with particles < 300 μm in 11/65 cases; CGD incidence was numerically higher among patients treated with particles < 300 μm than with particles > 300 μm (27.3 vs. 5.6%, *p* = 0.056), although this association did not reach statistical significance. 4/6 patients required cystoscopic removal of sloughed tissue. After resolution of the acute CGD episode, mean International Prostate Symptoms Scores (IPSS) and quality of life due to urinary symptoms (QOL) scores improved from 22 to 2.3 and from 5.3 to 0.5 respectively. In those with preserved ejaculatory function at baseline, 3/4 CGD patients developed new retrograde ejaculation.

**Conclusion:**

Central gland detachment is an uncommon but clinically significant complication of PAE. Severe prostatomegaly may be associated with CGD, and cases can occur despite use of medium-sized particles (> 300 μm). Long-term voiding outcomes are generally favourable, but new ejaculatory dysfunction can occur.

**Graphical Abstract:**

**Figure:**
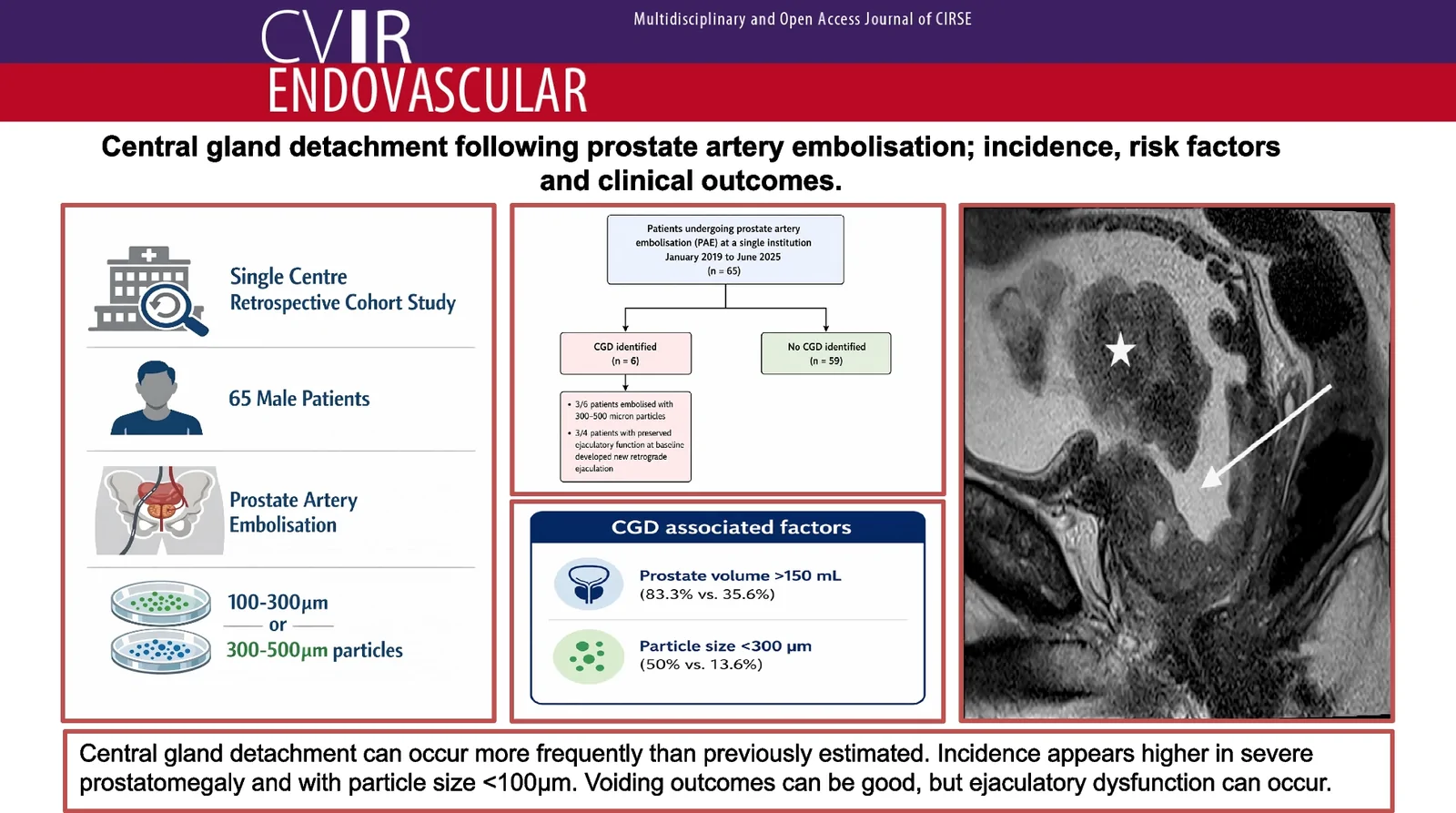

## Background

Prostate artery embolisation (PAE) is an established minimally invasive treatment option for patients with benign prostatic hyperplasia (BPH), as an alternative to transurethral resection of the prostate (TURP) [1]. Patients at increased perioperative risk or wishing to avoid TURP-related complications including sexual dysfunction are often referred for PAE [2, 3].

Central gland detachment (CGD) has been reported as a rare complication of PAE, and consists of fragments of necrotic prostate tissue becoming displaced into the bladder lumen [4–10]. These fragments can cause bladder outlet obstruction with urinary retention, pain and occasionally infection. A limited number of CGD case reports have been published (Table 1), and the largest published PAE cohorts suggest that this is a rare phenomenon (incidence less than 1%) [9, 11]. Hechelhammer et al. identified preprocedural catheter dependence, increased central gland index and increased post-procedural pain and inflammatory markers as independent risk factors, albeit based on just 3 cases among a cohort of 48 PAE patients with relatively mild prostatomegaly (mean volume 53 mL) [6]. Given the rarity of CGD and the limited published experience, reliable predictors of its occurrence and the optimal management of this complication remain undefined.

**Table 1:** Reported cases of central gland detachment to date

Author	Date	Journal	Cases	Particle size
Costa et al.	2016	JVIR	1	250 µ Embozene (Varian Medical Systems, Austin, Texas)
Leite et al.	2017	CVIR	3	100–300 µ Embospheres (± 300–500)
Ghelfi et al.	2018	CVIR	1	250 µ Embozene
Hechelhammer et al.	2019	JVIR	3	250 µ Embozene
De Assis et al.	2020	RB	1	Not confirmed for individual patient
Maqboul et al.	2021	JCU	1	300–500 µ Embospheres
Bhatia et al.	2025	JVIR	3	Not confirmed for individual patient

*JVIR *Journal of Vascular and Interventional Radiology, *CVIR *cardiovascular and interventional radiology, *RB *Radiologia Brasileira, *JCU *Journal of Clinical Urology

Sexual function is typically preserved after PAE but is at risk after the development of CGD [6, 12]. The necrosis that leads to CGD can result in defects in the prostate at the level of the bladder neck, similar to those seen following TURP [6]. Although the mechanism of retrograde ejaculation after PAE-induced CGD has not been studied directly, it may result from impaired bladder neck closure secondary to central gland necrosis and tissue loss, similar to the mechanism described following TURP [13, 14].

This single-centre retrospective cohort study aimed to define the incidence of central gland detachment and clinical outcomes following CGD, with a focus on voiding and sexual function. A secondary objective was to identify patient characteristics and procedural variables associated with increased risk of CGD.

## Methods

### Study design

A retrospective cohort study including all patients treated with PAE between January 2019 and June 2025 was performed. All procedures were performed in a single academic teaching hospital. Patients developing central gland detachment were identified and compared with the remainder of the cohort to explore potential clinical and procedural associations. This study was reported in accordance with the Strengthening the Reporting of Observational Studies in Epidemiology (STROBE) guidelines for cohort studies. The study was conducted in accordance with institutional policies. Due to the retrospective nature of this study, formal ethics committee approval was not required.

### Participants

All patients who underwent PAE were eligible for inclusion. All patients were males aged over 40, who had documented prostate volume > 40 mL on MRI or ultrasound imaging, and were referred for PAE after consultation with a urologist. Patients were referred for PAE due to persistent haematuria or due to moderate to severe obstructive lower urinary tract symptoms (LUTS) with IPSS score ≥ 8 and/or IPSS quality of life score ≥ 3. Those with or without an indwelling urinary catheter and with or without prostate cancer at baseline were included. Included patients with known prostate cancer either underwent PAE for LUTS in the setting of clinically insignificant prostate cancer (Gleason < 7), or persistent haematuria related to clinically significant prostate cancer. Patients with haematuria, indwelling catheters and known prostate cancer were included to reflect the patient heterogeneity present in many real-world PAE cohorts and to allow for subgroup analysis and identification of potential CGD risk factors.

Patients were excluded if embolisation was not performed or if at least one clinical follow-up appointment could not be confirmed.

### Procedures

PAE was performed after written informed consent with local anaesthesia and conscious sedation. All cases were performed in line with established techniques via common femoral artery access using a 5 F sheath with a 5-F catheter (most commonly Cobra C2, Cordis®) and a 2.4-F microcatheter (Direxion, Boston Scientific®). Cone-beam CT was routinely performed to identify prostatic arteries and prevent non-target embolisation. Intraarterial glyceryl trinitrate was administered into the prostatic arteries. Embolisation was performed with either 100–300 μm, 300–500 μm or a combination of 100–300 μm and 300–500 μm particles (Embospheres, Merit Medical Systems). During the study period, local practice changed to favour use of 300–500 μm particles as the initial embolic agent in most patients, based on published evidence suggesting increased adverse events with very small particles and the first cases of CGD in the institution [15, 16]. Bilateral embolisation was attempted in all cases. The target procedural endpoint was absence of prostatic enhancement on post-embolisation angiography and stasis of flow in the prostatic arteries. Following particle embolisation the prostatic arteries were coiled proximally with 2–3 mm coils (Azur, Terumo®) aiming to prevent vessel recanalisation.

### Definitions and outcome measures

Prostatic central gland detachment was diagnosed clinically based on solid material passing transcatheter or per urethra. In uncertain cases, including those patients presenting with urinary retention as their primary symptom, the diagnosis was confirmed via direct visualisation at cystoscopy or on MRI based on a combination of a new prostatic defect and free-floating prostatic tissue in the bladder lumen. MRI was performed selectively for symptomatic patients and not routinely following PAE.

Clinical baseline characteristics were compiled including prior surgical interventions, catheter dependence and clinical symptoms. Symptom severity was assessed before and after PAE with a standardised IPSS questionnaire and QOL score. In patients with an indwelling urinary catheter (in whom IPSS questionnaires are of limited utility), the most recent IPSS and QOL scores prior to catheter insertion were included. Patients who had experienced CGD were specifically asked about the presence of retrograde ejaculation or urinary incontinence.

Baseline prostate volume was calculated using the ellipsoid formula based on MRI [17]. In the absence of MRI, volume was calculated based on prostate ultrasound or procedural planning CT.

Data regarding clinical outcomes were gathered based on follow-up clinical consultations. This was routinely performed at 3 months following PAE with the performing Interventional Radiology (IR) physician. Any additional unscheduled IR and urology clinical assessments were also reviewed.

### Statistics

All analyses were performed on a per-patient basis. Descriptive statistics were used to summarise baseline characteristics, with continuous variables reported as means and standard deviations where appropriate. Prostate volume demonstrated numerical outliers and was therefore summarised using the median, interquartile range (IQR), and range, with between-group comparisons performed using the Mann–Whitney U test. In addition, prostate volume was dichotomised at 150 mL, to allow for exploratory subgroup analysis. This threshold was selected based on its prior use in the urology literature to define very large/massive prostate volumes in studies regarding BPH intervention [18, 19]. The categorisation was considered exploratory due to the small number of CGD cases, and was not intended to represent a validated threshold for risk prediction.

Categorical variables were compared using Fisher’s exact test. Normally distributed continuous variables were compared using the Student’s *t*-test, while the Mann–Whitney *U* test was used for continuous variables with non-normal distributions or influential numerical outliers. A two-sided *p* value < 0.05 was considered statistically significant.

## Results

### Study population

**Fig. 1:**
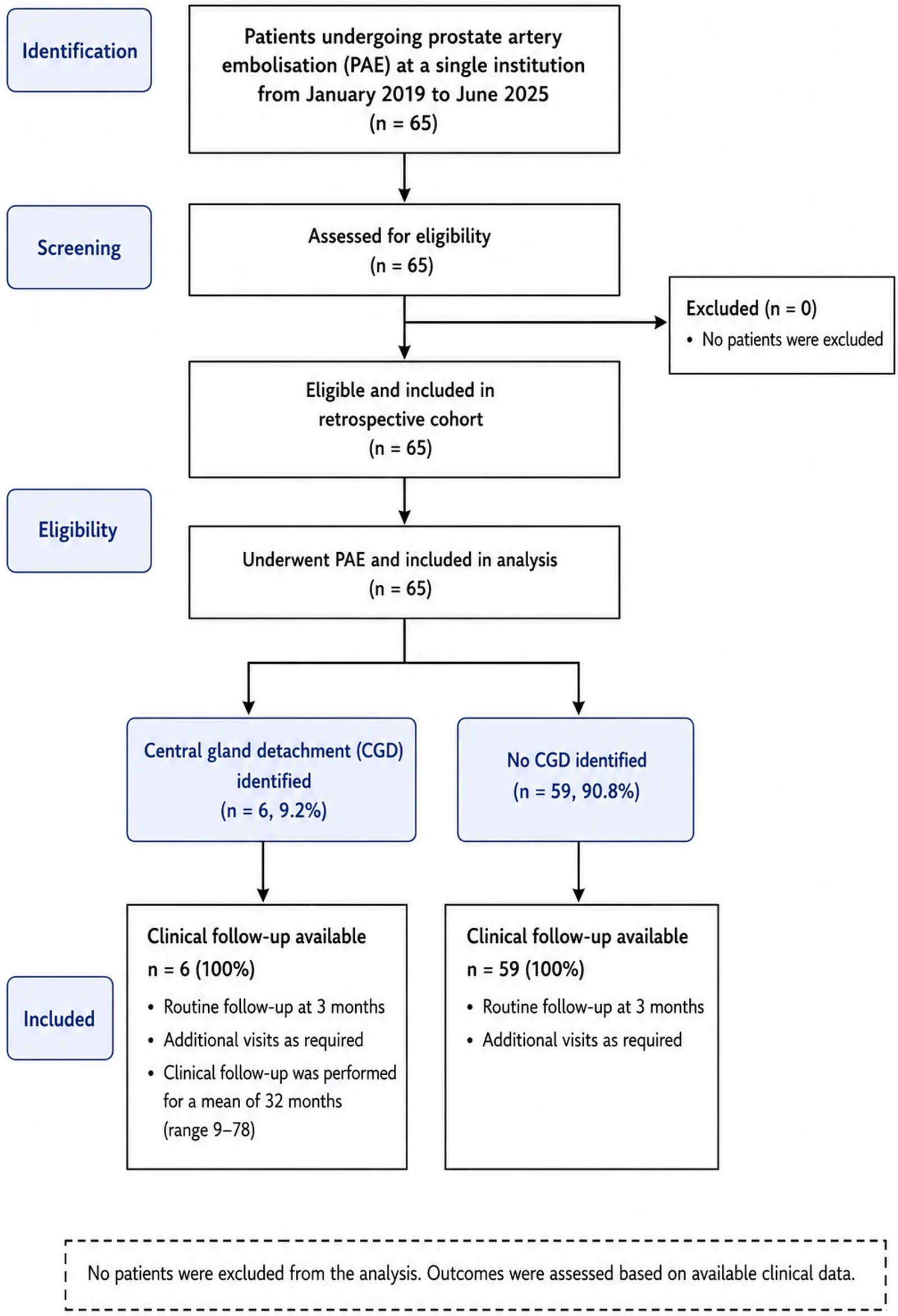
STROBE-style flow diagram of the cohort included in analysis

Between January 2019 and June 2025, 65 male patients underwent PAE. Patients were aged 49–93 years (mean 72 years). All patients met the specified inclusion criteria for analysis and no patient who underwent PAE during the study period was excluded (Fig. 1). Clinical follow-up data were available at 3 months or later for all patients.

The primary indication for PAE was BPH-related lower urinary tract symptoms in 56/65 patients (86.2%), while 9/65 (13.8%) underwent PAE for persistent haematuria. Prior to treatment, 28 patients had an indwelling urinary catheter (transurethral catheter, *n* = 25; suprapubic catheter, *n* = 3), and one additional patient performed self-intermittent catheterisation. Nine patients had a diagnosis of prostate cancer, including five with Gleason grade ≥ 7 disease. Previous transurethral prostate intervention had been performed in 11 patients (TURP, *n* = 9; Rezum™ water vapour therapy, *n* = 1; GreenLight™ laser therapy, *n* = 1).

Pre- and post-procedural IPSS and QOL scores were available for 38/65 patients (58.5%). Outcome scores were less frequently available in patients with indwelling catheters (6/28, 21.4%) and in those treated for haematuria (1/9, 11.1%), compared with the remaining cohort (31/34, 91.2%). Mean baseline IPSS and QOL scores were 22.4 and 5.3, respectively.

Bilateral embolisation was achieved in 61/65 patients (94%). Six patients underwent repeat PAE due to persistent or recurrent symptoms. Most patients (54/65, 83.1%) were treated exclusively with 300–500 μm Embosphere particles; the remaining 11 patients received 100–300 μm particles either alone or in combination with 300–500 μm particles. 9 of these 11 patients underwent PAE early in the study period (August 2022 or earlier), reflecting a change in local practice during the study period to favour use of particles > 300 μm in most patients.

### CGD incidence and associated factors

Six patients presented with signs and symptoms of central gland detachment following PAE (incidence 6/65, 9.2%). Mean clinical follow-up in these patients was 32 months (range 9–78). Patients reported passing strand-like solid material per urethra or through an indwelling catheter (5/6) and/or urinary retention (5/6), which was intermittent in 3/5 patients. One patient developed urinary infection after presenting with an obstructed suprapubic catheter and required high dependency unit admission.

Symptoms developed between 2 and 22 weeks following PAE. CGD was confirmed via cystoscopy in 4/6 and with MRI in 5/6.

Table 2 compares baseline characteristics and procedural factors relating to those who developed CGD and the remainder of the PAE cohort. Three of 6 patients who developed CGD were embolised using 100–300 μm particles, alone or in combination with 300–500 μm particles. The remaining three were embolised with 300–500 μm particles alone. Incidence of CGD in those embolised with 100–300 μm particles was 27.3% (3/11), compared to 5.6% (3/54) following embolisation with particles > 300 μm (*p* = 0.056). While overall prostate volume was not significantly different between those with and without CGD (178.5 vs. 146.9 mL respectively, *p* = 0.33), CGD occurred more frequently in patients with prostate volume > 150 mL compared with ≤ 150 mL (incidence 5/26 (19.2%) vs. 1/39 (2.6%), *p* = 0.03). All six patients who developed CGD had been referred for PAE for obstructive LUTS; those referred for PAE to treat haematuria did not develop CGD.

**Table 2:** Baseline characteristics and procedural factors

	Patients with central gland detachment (*n *= 6)	Patients without central gland detachment (*n* = 59)	*p *value
Age^a^	73.3 (± 11.6)	72.1 (± 9.0)	0.76
IPSS^a^	22 (± 3.6)	22.5 (± 7.1)	0.91
QOL score^a^	5.3 (± 0.58)	5.3 (± 0.97)	0.88
Prostate volume, mL^b^	174.5 (97, 183)	130 (117, 376)	0.22
Volume > 150 mL	5/6 (83.3%)	21/59 (35.6%)	0.03
Catheter-dependent	4/6 (66.7%)	24/59 (40.7%)	0.39
Prostate cancer	0/6	9/59	0.58
Prior TURP or other transurethral prostate intervention	2/6	9/59	0.27
Bilateral embolisation	6/6	55/59	1.00
Particles < 300 micron used	3/6	8/59	0.056

^a^Normally distributed continuous variables are presented as mean ± standard deviation

^b^Prostate volume is presented as median (interquartile range [IQR], range) because of the presence of marked numerical outliers

### CGD management and clinical outcome

Following diagnosis of CGD, 4/6 patients required cystoscopic removal of the sloughed prostatic tissue. The remaining two patients had resolution of symptoms with conservative management. No patient had evidence of bladder ischaemia on MRI (0/5) or cystoscopy (0/4).

**Fig. 2:**
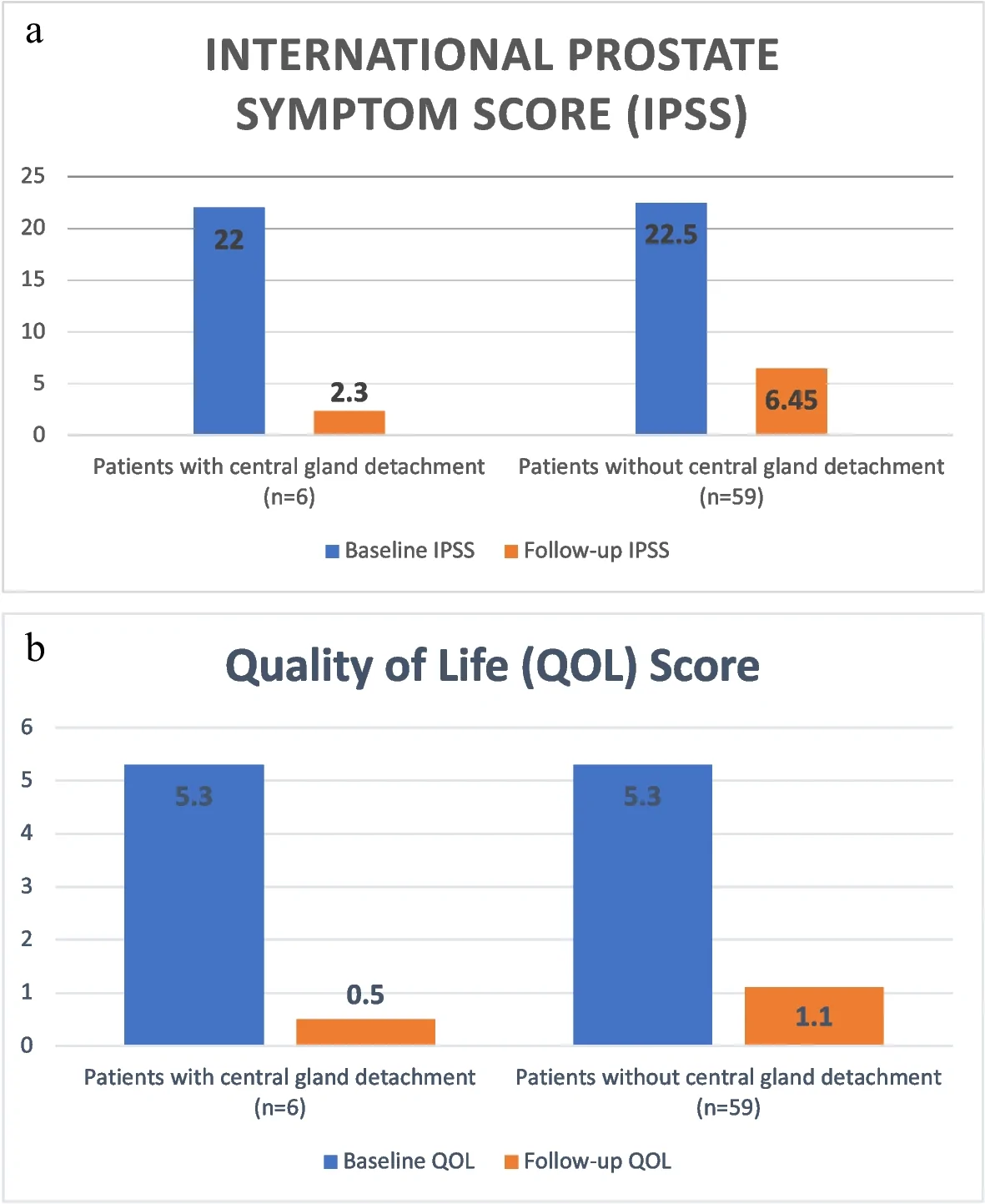
**a **Pre- and post-procedure IPSS for those with and without central gland detachment. **b **Pre- and post-procedure quality of life (QOL) scores for those with and without central gland detachment

In the four patients who were catheter-dependent at baseline, 2/4 remained catheter-dependent at follow-up. After resolution of the acute episode of CGD, the other 4 CGD patients all experienced an improvement in symptoms related to BPH, with average IPSS 2.3 post-PAE (compared to 22 prior to intervention, Fig. 2a). QOL score improved from 5.3 to 0.5 (Fig. 2b). All 4 were fully continent at follow-up.

**Fig. 3:**
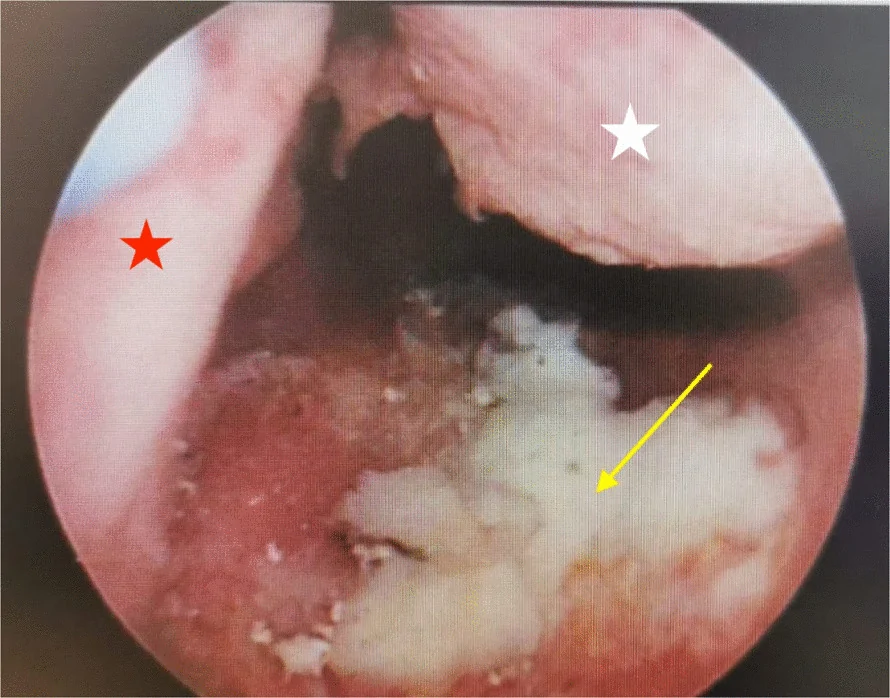
Cystoscopic image demonstrating a large prostatic defect at the level of the bladder neck due to sloughed tissue arising from median lobe *(yellow arrow)* and left lobe *(white star)*. The right prostatic lobe is intact *(red arrow).* There is no evidence of bladder wall ischaemia or necrosis

**Fig. 4:**
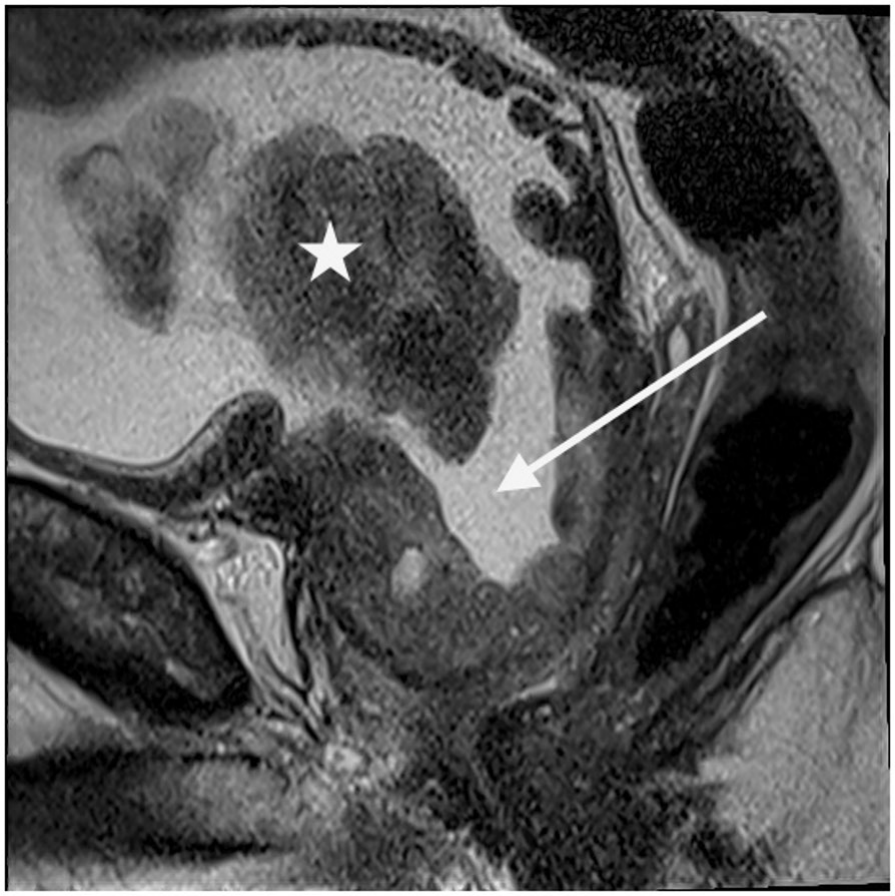
MRI Prostate performed on a different patient 6 weeks after PAE. A new prostatic defect is present at the bladder neck (white arrow). A large volume of sloughed prostatic tissue (white star) is noted within the lumen of the urinary bladder

Information regarding sexual function and continence was available for 5/6 patients who experienced CGD (one patient could not be contacted to provide follow-up data). 3/4 patients with preserved ejaculatory function at baseline developed new retrograde ejaculation following PAE. These patients all had a large prostatic defect at the bladder neck on follow-up MRI (example Figs. 3 and 4).

## Discussion

In this single-centre retrospective cohort study, central gland detachment was found to be a commonly encountered complication of prostate artery embolisation, with an estimated incidence of 9%. Baseline prostate volume over 150 mL was associated with a higher rate of CGD, while there was a trend toward increased CGD incidence using particles < 300 μm. While spontaneous resolution of symptoms was possible, the majority of patients required cystoscopic removal of sloughed prostatic tissue. After resolution of the acute episode, mean IPSS and QOL scores dramatically improved from 22 to 2.3 and 5.3 to 0.5 respectively. The majority of patients experiencing CGD developed new retrograde ejaculation.

The present findings add further nuance to the understanding of CGD, which was previously thought to be solely a consequence of aggressive embolisation with very small particles [5, 12, 20]. This cohort demonstrates that CGD may also occur following embolisation with medium-sized particles (> 300 μm), with three such cases observed. Based on the reported literature, this represents the largest reported series of CGD occurring after embolisation with particles > 300 μm. Furthermore, the first evidence of a possible link to overall prostate volume is presented, with an increase in CGD incidence suggested in very large prostates (prostate volumes exceeding 150 mL). Hechelhammer et al. [6] did not identify a link between overall prostate volume and CGD, though mean prostate volume in their cohort was relatively low (53 mL vs. 150 mL in the present cohort). The relatively high incidence of CGD in this cohort (9%) may be explained by the high mean prostate volume, with the highest observed incidence occurring in prostates exceeding 150 mL. While the mean prostate volume of 150 mL exceeds that reported in many published PAE cohorts (in which mean prostate volumes of 83–107 mL have been reported) [9, 11, 21], it reflects a clinically important population with severe prostatomegaly and limited alternative treatment options for LUTS, and may better represent the real-world referral patterns encountered in developing PAE services. The high proportion of catheter-dependent patients (43%) may also have contributed to the observed incidence.

No other reliable clinical or procedural predictors of CGD were identified. However, the only patient in the cohort who had undergone prior Rezum™ water vapour therapy before PAE subsequently developed CGD. Cases of CGD have been reported following Rezum™ therapy alone [22]*.* With the increasing use of both PAE and minimally invasive surgical therapies (MIST) for BPH, more patients are likely to present for PAE after prior prostate intervention. Thermal therapies such as Rezum™ induce central gland necrosis and remodelling, which may theoretically increase susceptibility to CGD when PAE is subsequently performed. Further study in larger cohorts is warranted, particularly in patients with prior MIST, to better define this potential risk.

Conventional PAE practice has generally favoured aggressive embolisation to reduce revascularisation and symptom recurrence; described techniques include slow particle injection, vasodilator use and PErFecTED embolisation [23, 24]*.* The embolisation strategy adopted by the operators in this study was in line with these conventional techniques. An alternative perspective (proposed by Bilhim et al.) emphasises preserving PAE as a low-morbidity treatment and advocates less aggressive embolisation to reduce complications such as CGD [12, 20]*.* Rather than applying a universal strategy, operators should individualise embolisation technique according to patient characteristics and treatment goals.

When assessing complications following PAE, the available treatment alternatives should also be considered. At the study institution, patients are referred for PAE only after urological assessment and discussion of alternative management options. Many patients with very large prostates had been considered unsuitable for TURP (typically recommended for prostates up to approximately 80 mL [25, 26]) and/or carried prohibitive operative risk for prostatectomy. In selected patients, a staged pathway involving PAE followed by cystoscopic tissue removal may represent an acceptable outcome, particularly where relief of LUT-related symptoms is prioritised and patients are counselled regarding the possibility of ejaculatory dysfunction. Similar staged management strategies are accepted in uterine fibroid embolisation following appropriate pre-procedural counselling [27]. These findings highlight the importance of multidisciplinary discussion and ongoing collaboration between interventional radiology and urology services.

De-novo retrograde ejaculation occurred in 3/4 patients with CGD and preserved ejaculatory function prior to PAE. This is consistent with prior CGD cohort study data [6] and adds to a growing weight of evidence that ejaculatory dysfunction is possible after PAE; quoted rates of dysfunction following PAE include 12% in a retrospective cohort study [28], 24% among the UK ROPE trial participants [21] and 56% in a Swiss randomised controlled trial [1]. Although less common and generally less severe than after TURP [1, 29], ejaculatory dysfunction post-PAE should not be understated. Better identification of CGD predictors will allow interventional radiologists to find a balance between achieving a good voiding outcome and preserving ejaculation, tailoring their embolisation strategy to the clinical outcomes prioritised by the individual patient.

This study had a number of limitations. All PAEs were performed under a single operator in a single academic hospital, which may limit generalisability. In particular, prostate volumes in those undergoing PAE may be lower in many other centres, and this may have implications for local CGD incidence. Analysis of potential risk factors was based on just six cases of CGD, raising the possibility that any observed associations may have been due to chance. The volume of particles used during PAE was not recorded prospectively; however, embolisation endpoints used in this cohort were similar to those reported in the literature. CGD identification was retrospective and routine post-PAE MRI was not performed, with imaging or cystoscopy only requested in the presence of suspicious symptoms. Subclinical cases may have been missed, potentially leading to the true incidence of CGD being underestimated. The true incidence of ejaculatory dysfunction in the cohort was not evaluated, as only those with CGD were questioned. IPSS/QOL scores were only available for 58% of the cohort due to a large proportion of catheter-dependent patients in whom scores were often unavailable. Finally, all embolisations were performed with particles (Embospheres, Merit Medical Systems), and as such the risk of CGD with glue or other embolic agents was not evaluated.

## Conclusion

This retrospective cohort suggests that central gland detachment following prostate artery embolisation may occur more frequently than previously recognised, particularly in patients with severe prostatomegaly. The observed incidence was higher in patients with prostate volumes > 150 mL, although this association was exploratory and requires confirmation in larger cohorts. Incidence may also increase with use of particles < 300 μm, although cases occurred with larger particles. While cystoscopic tissue removal was frequently required, observed voiding outcomes were generally favourable; however, new ejaculatory dysfunction can occur.

## Data Availability

The datasets used and/or analysed during the current study are available from the corresponding author on reasonable request.

## Abbreviations

CGD: Central gland detachment
PAE: Prostate artery embolisation
IPSS: International prostate symptom score
QOL: Quality of life due to urinary symptoms
BPH: Benign prostatic hyperplasia
TURP: Transurethral resection of the prostate
LUTS: Lower urinary tract symptoms
MIST: Minimally invasive surgical therapies
STROBE: Strengthening the Reporting of Observational Studies in Epidemiology
